# Supplementary material for: Flooding and ecological restoration promote wetland microbial communities and soil functions on former cranberry farmland
Source: PLoS One. 2021 Dec 17;16(12):e0260933. doi: 10.1371/journal.pone.0260933 (PMC8683025; doi:10.1371/journal.pone.0260933)
Supplement: S1 File — (DOCX) [file pone.0260933.s002.docx]

Supporting Information for Rubin et al. 2021

**Title:** Flooding and ecological restoration promote wetland microbial communities and soil functions on former cranberry farmland

**S1 Fig** Representative photos of sites. This composite figure is modified with permission of the authors, from Ballantine et al. 2020, “Learning from the restoration of wetlands on cranberry farmland: preliminary benefits assessment. Living Observatory, 2020.

**S2 Fig** We tested the sensitivity of our sampling scheme. The original standard deviation from the full dataset is shown as grey diamonds, and the new standard deviations are shown in color. For the three combinations of dropping two sites from young retired (Tidmarsh East 1, Tidmarsh East 2, and Tidmarsh East 3), standard deviation for this category was more likely to decrease, as indicated by colored points falling below the diamond for the majority of cases. For the restored sites, there were nine combinations of dropping two sites at a time from Eel River N, Eel River S, Eel River NE, and two sites at a time from Tidmarsh W 1, Tidmarsh W 2, and Tidmarsh W 3. Again, for the majority of cases, dropping sites decreased variation within this category, rather than increasing it. Together, this validates our sampling and treatment of all 23 sites as independent.

**Detailed soil analyses methods**

A total of 36 physicochemical analyses were performed on the 184 soil samples. 21 of these variables including macronutrients and micronutrients, along with pH, SOM, scoop density, cation exchange capacity, base saturation of Ca, Mg, and K, were performed by the UMASS plant and soil testing lab using standard protocols. Methods for the remaining 15 variables are described below.

Soil moisture was determined gravimetrically for each sample using the difference in mass between oven-dried soil (105°C until constant weight) and field-moist soil. Bulk density was determined by dividing total dry weight of each soil sample by volume of the core.

Denitrification potential was determined using assays of denitrification enzyme activity (Groffman et al., 1999) within 48 hours of collection. This method adds an abundance of carbon and nitrate to each sample in an oxygen free container so that nitrogen gas production via denitrification is a function only of the amount of enzyme present (Groffman et al., 1999). In our assays, acetylene gas was injected into sealed 125-mL flasks containing 5 g of dry soil and 10 mL of liquid media (0.72 g L^-1^ KNO_3_ and 0.5 g L^-1^ glucose to provide substrate for enzymes, plus 0.125 g L^-1^ chloramphenicol to block further enzyme production). Flasks were flushed with N_2_ gas to remove oxygen from the headspace prior to injection of acetylene. Acetylene inhibits the reduction of N_2_O to N_2_, and as such, the product of denitrification in this assay is N_2_O. Soil slurries were mixed constantly for 90 minutes on an orbital shaker. Gas samples were taken from the soil slurries at time 30 and 90 minutes, injected into evacuated 9 mL gas vials, and analyzed for N_2_O using a Shimadzu GC-14 gas chromatograph equipped with an electron capture detector. Denitrification potential (rates) were determined by regression of N_2_O accumulation against time.

Microbial biomass C and N content were measured using the chloroform fumigation–incubation method (Jenkinson and Powlson, 1976). Microbial cells in the soil samples were killed and lysed by fumigation for 20 hr with chloroform. The fumigated samples were then inoculated with a small amount of fresh soil, introducing microorganisms that metabolize the lysed microbial cells in the original sample. The CO_2_ and extractable mineral N (NH_4_^+^ and NO_3_^−^) released by the actively-growing cells were measured during a ten-day incubation. The CO_2_ and extractable mineral N detected in this assay are directly proportional to the amounts of C and N that were present in the microbial biomass of the original soil.

Ten-day incubations of non-fumigated samples provided measurements of potential net mineralization and nitrification rates, as well as greenhouse gas emissions. Potential net N mineralization was quantified by measuring the accumulated mineral N (NH_4_^+^ and NO_3_^−^), and potential net N nitrification was quantified by measuring the accumulation of NO_3_^−^ during this incubation. Extractable soil NH_4_^+^ and NO_3_^−^ were quantified colorimetrically using a Lachat Quikchem 8100 flow injection analyzer (Hach Company, Loveland, CO, USA). At the end of the 10 day incubation, production of CO_2_ was measured on a Shimadzu GC-14 gas chromatograph with a thermal conductivity detector (Shimadzu, Kyoto, Japan).  Potential net methane emissions were measured from these same samples on a Shimadzu GC-8 gas chromatograph with a flame ionization detector.  We analyzed N_2_O using Shimadzu GC-14 with electron capture detector.
