# Supplementary material for: Flooding and ecological restoration promote wetland microbial communities and soil functions on former cranberry farmland
Source: PLoS One. 2021 Dec 17;16(12):e0260933. doi: 10.1371/journal.pone.0260933 (PMC8683025; doi:10.1371/journal.pone.0260933)
Supplement: Attachment — Submitted filename: Response to Reviews_1.19.21.docx [file pone.0260933.s003.docx]

Rachel Rubin, PhD

Mount Holyoke College

Environmental Studies

South Hadley, MA 01075

720-415-7709

January 22, 2021

Dear Editorial Board,

We would like to resubmit our revised manuscript, “Flooding and ecological restoration promote wetland microbial communities and soil functions on former cranberry farmland” to PLOS ONE for consideration in the *Restoration and Rewilding Special Issue.*

We are grateful for the contributions of the reviewers and the editor, and hope that the manuscript can now be accepted for publication.

In the following pages you will find our response to reviewers, as well as our revised manuscript.

Sincerely,

Rachel Rubin

Kate Ballantine

Arden Hegberg

Jason Andras

We note that you have provided funding information that is not currently declared in your Funding Statement. However, funding information should not appear in the Acknowledgments section or other areas of your manuscript. We will only publish funding information present in the Funding Statement section of the online submission form. Please remove any funding-related text from the manuscript and let us know how you would like to update your Funding Statement. Currently, your Funding Statement reads as follows:  [Joint Genome Institute Project 1188787] Please include your amended statements within your cover letter; we will change the online submission form on your behalf.

RR et al: Please change funding statement, on our behalf to: Funding was provided by Joint Genome Institute Project 1188787. The funder had no role in study design, data collection, decision to publish, or preparation of the manuscript.

Thank you for stating the following in your Competing Interests section: **[NO].** Please complete your Competing Interests on the online submission form to state any Competing Interests. If you have no competing interests, please state “The authors have declared that no competing interests exist.”, as detailed online in our guide for authors at <http://journals.plos.org/plosone/s/submit-now>**.** This information should be included in your cover letter; we will change the online submission form on your behalf.

RR et al: Please change the form, on our behalf, to say: The authors have declared that no competing interests exist.

4) We note that Figure 1 in your submission contain [map/satellite] images which may be copyrighted. All PLOS content is published under the Creative Commons Attribution License (CC BY 4.0), which means that the manuscript, images, and Supporting Information files will be freely available online, and any third party is permitted to access, download, copy, distribute, and use these materials in any way, even commercially, with proper attribution. For these reasons, we cannot publish previously copyrighted maps or satellite images created using proprietary data, such as Google software (Google Maps, Street View, and Earth). For more information, see our copyright guidelines: <http://journals.plos.org/plosone/s/licenses-and-copyright>.

RR et al: Figure 1 was obtained from stamen maps, http://maps.stamen.com/#watercolor/12/37.7706/-122.3782. Stamen maps, and the underlying data, are produced under a creative commons license. We added the following text to Fig 1 legend: Map tiles by Stamen Design, under CC BY 3.0. Data by OpenStreetMap, under ODbL.

Reviewer #1: PONE-D-2033816
The manuscript is generally written very clearly and concisely. I am very grateful to the authors for that! The subject matter is also very interesting. Unfortunately, as the manuscript currently reads, I cannot recommend publication because 1) the analyses reflect pseudoreplication, 2) the soil sampling may have been flawed, and 3) age differences among sites of different treatments may prohibit comparisons among treatments.

Pseudoreplication
Lines 118-123. As far as I can tell, there is a single reference wetland, 2 actively restored sites, 3 passively restored sites, an unknown number of retired sites and an unknown number of active farms. The authors need to be clearer about replication for each treatment. This information and site descriptions should probably be made available in a table. Despite the limited replication at the site level, each individual soil sample was apparently analyzed as if it were independent of the other samples. I do not see how the authors can justify this pseudoreplication. The unit of replication is the SITE, not the SOIL SAMPLE because the treatments were applied at the level of the site, not the level of the soil sample. Therefore, these analyses are incorrect.

RR et al: We agree that our handling of sample replication could be clarified and improved. To this end:

- We clarified site replication as follows in L279: “Treatments included: cranberry farm (3 sites), young retired (5 sites), old retired (3 sites), flooded (3 sites), restored (6 sites), and natural (3 sites). Natural reference sites have no history of cranberry farming (Fig 1).”
- Following their introduction in the abstract, we removed references to broad geographic features such as “wetlands” or “farms” and instead constrain our language to “sites” throughout the manuscript.
- We added the 23 site labels to the map (Fig 1), so that they can be matched to the site descriptions that are provided in the excel sheet in the Supplementary Material.
- We updated the analysis as well as the presentation of Fig 2 to reflect the original sample replication, rather than all possible pairwise comparisons between a given sample and a given natural wetland sample. For example, replication for similarity to a natural wetland, for the farm category, went from N=504 to N=24. The resultant analysis is much more conservative, but overall conclusions did not change.
- To justify our choice of treating all 23 sites independently, we performed a new sensitivity analysis to justify our sampling.

See L 363:Some areas on the map (Fig 1) contained multiple sampling sites. We did this in order to capture variation within particularly large restoration projects (Tidmarsh West and Eel River), or because sites co-occurred with other long-term monitoring objectives (Tidmarsh East). To validate that our sampling captured more variation across these areas, we conducted a sensitivity test by systematically dropping two sites from Tidmarsh West, Eel River and Tidmarsh East (S2 Fig in S2 File). For the majority of cases, dropping two sites at a time from the Tidmarsh East 1, 2, and 3, Tidmarsh West 1, 2, and 3 and Eel River North, Northeast, and South was more likely to decrease variation than to increase variation. Therefore, we treated all 23 sampling sites as independent throughout the analysis.

Soil sampling
Line 140. If the ratio of muck to sand varies from site to site, then one will probably see corresponding variation in microbial community structure, SOM concentration, CEC, etc. that has more to do with the relative amounts of muck and sand rather than anything to do with restoration effort. In other words, the properties of the muck may be highly similar in ALL sites, and the differences and similarities among sites may be explained simply by the amount of sand vs. muck in the soil samples, not necessarily by the level of restoration.

RR et al: SOM accumulation, is, in fact a direct outcome that the treatments seek to maximize, and is not a confounding variable. All cranberry farms start out sandy, and the goal of this study was to track the development of SOM as well as microbial communities. To this end, we added the following new text to the first paragraph of the discussion: “Restoration success is often defined by the convergence of biological and functional variables towards reference conditions. A principal goal of ecological restoration on former cranberry farmland is to promote soil organic matter development; by filling in existing ditches, breaking up the deep sand layer using surface grading, and reconstructing a meandering stream channel, soil organic matter can begin to accumulate. We interrogated this process of soil development by comparing restoration to the simple action of leaving the cranberry farms in the ‘harvest condition’—permanently flooded, using wooden boards.”

Age effects
Lines 118 – 123. Obviously, it is difficult to find retired farms, passively restored sites, and actively restored sites that are similar to each other except in the way they have been managed following farm retirement. But clearly time may affect many of the variables that were assessed. There are 3 replicate passively restored sites of the same age (4 y), but the actively restored sites were 1 and 6 years old, and the retired farms were newly retired (1-4 y) or “old retired”: (17-20 y). Which of the active farms, which of the actively restored sites, which of the retired farms, and which of the passively restored sites can logically be compared to the reference wetland? The authors will have to take age into consideration in drawing conclusions. On line 244, the authors remove site age as a “nuisance variable”. I do not understand the justification for doing so. I do not see how you can remove the effect of age from among, for example, the actively restored sites, of which there are only two. Presumably every variable is modeled as a linear function of age, but how can that be justified if there are only two replicates of given treatment? Moreover, there is but a single reference wetland. Such a justification is possible only if each soil sample is considered an independent variable, and I have already indicated above why this is not so.

RR et al: As the reviewer noted, it’s not possible to model age as a predictor of similarity, because there are not enough age classes in our dataset to ask this particular question. We do agree that the role of age can be made more explicit, though. Therefore, we split up the retired sites into “old retired” and “young retired”. In this way, it becomes more clear that the young retired sites and the old retired sites are highly similar to one another, indicating retired sites don’t achieve wetland status, regardless of their age. This analysis was previously provided in the supplementary material. The young retired, flooded, and restored sites are all relatively young (less than 6 years), and are now roughly comparable to each other. Furthermore, we did not include age from the capscale analysis in this revision (it was originally coded as a conditional effect, or ‘nuisance’ variable). There was no change to our conclusions.

Smaller editorial comments:
Line 102. It is not immediately clear what the difference is between retired farms and flooded sites because flooded sites are presumably retired farms.

RR et al: see L209: “Rather than allowing retired farms to develop into upland forest, some retired farms are instead kept flooded using wooden boards.”

Lines 101 – 103. Are these really the only questions? Certainly, these are important but, judging from the rest of the introduction, an important underlying management question concerns the effectiveness of passive flooding as a restoration strategy. Shouldn’t that be included?

RR et al: That is correct. We revised the results subheadings so that they are now the same three research questions. This should improve clarity.

Line 106. Yes, but what basis is there for thinking that simply because “extensive action” was taken, active restoration would be the best strategy? Is there any evidence for this expectation? One could possibly propose another even more logical, opposing hypothesis, which is that active restoration would result in less SOM accumulation because of the greater level of disturbance.

RR et al: Revised to: “We expected that denitrification potential and soil organic matter would be important for explaining variation in microbial communities across the six treatment categories, and that these outcomes would be highest at wet sites (natural, restored, and flooded) and lowest at the drier sites (farm, young retired, and old retired).”

Line 115. Describing the length of the perimeter is not helpful at all unless we know the shape.

RR et al: See the map (Figure 1), which shows the shape of the points surveyed; perimeter and area were calculated from the same outline created by these points.

Lines 128-129. Previously the authors used the term “flashboard”, but here they use the term “wooden board”. Keep it simple and use just one term.

RR et al: Changed to wooden boards throughout.

Line 141. “…at a depth of 10 cm” is not clear. Do the authors mean “…to a depth of 10 cm”? What does “Soil samples (50 cm3)” mean? Does this mean that 50 cm3 samples were taken at 10 cm depth? Were all 8 samples homogenized together, or do you mean each of the 8, 50 cm3 samples was homogenized?

RR et al: “Plants and mosses were removed from the surface, and soils were collected to a depth of 10 cm and a width of 5 cm. Soil samples were homogenized in their own bags and subsampled in the field…”

Reviewer #2: The manuscript presented by Rubin et al is an interesting example of how restoration processes have effects on microbial soil communities. The paper is well written, and I think it is easy to understand. I agree with the conclusions obtained here; however, I have some concerns about the data showed here and the data that the authors did not show.

Please, show at least in supplementary data, a table containing number of raw reads, reads per sample after filtering, taxonomy… those data showed in most of papers, that I believe is mandatory.

We appreciate the reviewer’s suggestion to include sequencing depth and number of taxa in the main text. The main text now reads:

Line 416: “denoising was performed separately on each run, resulting in 22,176,127 total sequences (**an average of 129,857 sequences per sample**), and 13,978 unique ASV’s across 183 samples (**an average of 1,579 ASV’s per sample**).”

Line 427: “For 18S amplicons, forward and reverse reads were trimmed to 144 bp and denoised, resulting in 11,430,013 total sequences (**average of 36,859 sequences per sample**) amounting to 70,836 unique ASV’s across 183 samples (**average of 407 ASV’s per sample**).”

Authors refer ASVs correspond to unique taxonomic assignments to genus and species level for 16S (lines 173-175). Which genera within the identified Classes are the most abundant? Same question remains unanswered for microeukaryotic populations.
RR et al: Since our goals were to understand which treatments resulted in similar communities to wetlands, and to identify indicators of treatments (which we conducted at the class level for Figs 4 and 5),we do not feel that reporting the most abundant genera would have contributed to our analysis, not to mention there are 600-900 genera in each dataset. However, please note that in Fig 6 we draw attention to the two most abundant micro-eukaryote genera, which were also among the most responsive to treatments.

How the authors removed chimeras and sequences from plants/algae in the 16S analyses?
RR et al: Chimeras are removed in the denoising step in the QIIME2 workflow, using DADA2. See line 410: “Amplicon sequence variants (ASV’s) were identified using the Divisive Amplicon Denoising Algorithm (DADA2), which merges sequences, removes chimeric sequences, removes reads with ambiguous bases, and corrects sequence errors so that amplicon sequence variants can be resolved to the level of single-nucleotide differences (Callahan et al. 2017). There are no plant contaminants in the 16S analysis. There are chloroplast and mitochondrial contaminants in 16S analysis, which were removed in the filtering step (Line 429).

Some minor comments:
Line 74, remove one ditches

RR et al: Thank you

Line 149-154, please identify the regions for 16S and 18S (V3-V5, V4…)

RR et al: Text now reads: “For prokaryotic amplicons, libraries were prepared by amplifying the **V4-V5 hypervariable region** of the 16S rRNA gene…” and “..libraries were prepared by amplifying the **V9 hypervariable region** of the 18S rRNA gene..”

The elimination of the R2 raw reads represents here an issue. Please, explain pros and cons of using only R1 or give more reasons/discussion about how this affects the data quality. This is a limitation of the study.

RR et al: Due to the difference in quality between forward and reverse reads, it made more sense to drop the reverse reads. Merging them would have depleted the quality of our dataset rather than improve it. This is common for this particular gene region, and we added this note to the full text in L 177.

Data regarding to sequencing must be presented at least as supplementary data.

RR et al: We updated the Supplementary Data File. As requested, sequence counts are now provided as two additional columns entitled “16S #sequences” and “18S #sequences”.

Line 181, Charophytes refers only to green algae, why the authors remove this taxon if they form part of the microbial communities as a whole?

RR et al. Since charophytes are plant ancestors, they are classified D_3_charophyta in the SILVA database. In 13 out of 15 cases, charophyta referred to land plants (e.g. Gallium, Poaceae), and in only two cases (two ASV’s), charophyta corresponded to green algae.

Please, upload raw reads to SRA NCBI for better data accessibility. MG-RAST showed no results for the number provided.

RR et al. Sequences are now provided on the NCBI sequence read archive (SRA), under accession #PRJNA693513
