# Supplementary material for: Flooding and ecological restoration promote wetland microbial communities and soil functions on former cranberry farmland
Source: PLoS One. 2021 Dec 17;16(12):e0260933. doi: 10.1371/journal.pone.0260933 (PMC8683025; doi:10.1371/journal.pone.0260933)
Supplement: Attachment — Submitted filename: Response to Reviews PONE-D-20-33816.docx [file pone.0260933.s004.docx]

PONE-D-20-33816R1
Flooding and ecological restoration promote wetland microbial communities and soil functions on former cranberry farmland
PLOS ONE

Dear Dr. Rachel Rubin,

Thank you for submitting your manuscript to PLOS ONE. After careful consideration, we feel that it has merit but does not fully meet PLOS ONE’s publication criteria as it currently stands. Therefore, we invite you to submit a revised version of the manuscript that addresses the points raised during the review process.

Please submit your revised manuscript by 06.05.2021. If you will need more time than this to complete your revisions, please reply to this message or contact the journal office at [plosone@plos.org](mailto:plosone@plos.org). When you're ready to submit your revision, log on to <https://www.editorialmanager.com/pone/> and select the 'Submissions Needing Revision' folder to locate your manuscript file.

Please include the following items when submitting your revised manuscript:

- A rebuttal letter that responds to each point raised by the academic editor and reviewer(s). You should upload this letter as a separate file labeled 'Response to Reviewers'.
- A marked-up copy of your manuscript that highlights changes made to the original version. You should upload this as a separate file labeled 'Revised Manuscript with Track Changes'.
- An unmarked version of your revised paper without tracked changes. You should upload this as a separate file labeled 'Manuscript'.

If you would like to make changes to your financial disclosure, please include your updated statement in your cover letter. Guidelines for resubmitting your figure files are available below the reviewer comments at the end of this letter.

If applicable, we recommend that you deposit your laboratory protocols in [protocols.io](http://protocols.io/) to enhance the reproducibility of your results. Protocols.io assigns your protocol its own identifier (DOI) so that it can be cited independently in the future. For instructions see: <http://journals.plos.org/plosone/s/submission-guidelines#loc-laboratory-protocols>. Additionally, PLOS ONE offers an option for publishing peer-reviewed Lab Protocol articles, which describe protocols hosted on [protocols.io](http://protocols.io/). Read more information on sharing protocols at <https://plos.org/protocols?utm_medium=editorial-email&utm_source=authorletters&utm_campaign=protocols>.

We look forward to receiving your revised manuscript.

Kind regards,

Luigimaria Borruso
Academic Editor
PLOS ONE

Reviewers' comments:

Reviewer #1: PONE-D-20-33816R1

In general, this manuscript is very much improved over the first iteration. My main concern is Table 1, which appears to list the wrong values for n. I hope it is just an oversight and that the authors did not really perform incorrect analyses. In addition, the following are some other things to consider.

RR et al: The sample sizes in Table 1 are correct. As noted in the methods section, we collected 8 samples from each site. Each category had different numbers of sites. We added to the Table 1 figure caption; “N is the total number of samples across all the sites in each category”.

Line 68. “Data” is a plural word. Thus, “data that are…” is correct.
RR et al: addressed.

Lines 99-101. I do not understand the reasoning here. If flooding produces comparable results to restoration, why use flooding merely as an intermediate step, or as an alternative only when restoration is NOT an option? If it is comparable, why not use it ALL the time, particularly if it is easier, faster and cheaper than restoration?
RR et al: We revised the introduction to better reflect the chronology of this process, which is that retirement comes first, followed by land purchases/easements, then to flood or not to flood, followed by full restoration.

Line 120. I do not seem to have access to the S1 file. I looked for this file because I do not see a good description of the natural sites. In fact, they are not described as wetlands in the text, only that “natural reference sites have no history of cranberry farming (Fig 1).”

RR et al: Supplementary file S1 now has a representative photo for all six treatments, including natural wetlands.

Line 144. What does this mean: “For the MAJORITY of cases”? It might be better to state how many cases out of how many cases so the reader can make the judgement as to whether the procedure justifies what was done.
RR et al: line 154 now reads “8 out of 12 cases”

Line 154. “spaced 30 m apart” is not clear. In a straight line? Often non-random sampling schemes like this are not easily justified. The point is for the sampling to best represent the kind of real variability likely to exist at the site.
RR et al: corrected to “Eight soil samples (dysic, mesic Typic Haplosaprists of the Freetown series), were randomly selected at each site, and spaced 30 m apart.

Line 155. What kind of sampling device was used? It seems like a cylindrical soil probe was used, in which case the width should be given as the diameter.
RR et al: corrected to: “soils were collected using a 1.5 cm diameter soil corer to a depth of 10 cm, and flame sterilized between each sample”

Lines 233-234. How did you control for spatial autocorrelation?
RR et al.: We tested for spatial autocorrelation, but we didn’t control for it. Sentence now re-worded for clarity, from “There was no relationship between geography and soil variables, thus eliminating this source of autocorrelation” to “There was no relationship between geography and soil variables.”

Lines 234-235. It is probably more accurate to write “spatial correlation is unavoidable in many ecological studies”. But this still does not eliminate the potential problems interpreting this dataset. The interpretations have to take this potential confounding into consideration.

RR et al.: we further addressed this as a new paragraph in the discussion (paragraph two), as well as a new sensitivity test in the supplementary material. Our interpretation takes the potential confounding effects of spatial autocorrelation into consideration.

Line 242. Explain ASV.

RR et al: this is explained in the following sentence: “Amplicon sequence variants (ASVs) were identified using the Divisive Amplicon Denoising Algorithm (DADA2), which merges sequences, removes chimeric sequences, removes reads with ambiguous bases, and corrects sequence errors so that amplicon sequence variants can be resolved to the level of single-nucleotide differences (Callahan et al. 2017).” A reference is also provided for more details on the rationale for ASV’s.

Reviewer #3: The present article investigates the effects of retiring cranberry farms, and compares how retiring alone, flooding and active restoration are affecting soil microbial communities. Although numbers of real replicates are low, appropriate efforts such as statistical power tests have been applied to compensate for this drawback. Moreover, individual soils have been sampled intensively to account for within-soil heterogeneity. Certainly, it is not easy to find and sample suitable sites for these five(!) treatments. The ecological conclusions are important for restoration politics and can contribute to a site melioration during the time leading up to restoration.
I do have some minor points that should be addressed before publication:

INTRODUCTION

Do soil variables explain microbial communities or vice versa? You mentioned this point that is especially true for denitrification in the discussion (l445) but I suggest to pronounce it more in the introduction.
RR et al: It is indeed a chicken-and-egg question. See new revised text in the introduction: “The physical soil environment limits the establishment of microbial communities, which in turn influence their physical environment throughout wetland development. Despite this clear feedback loop, it is still unclear whether discrete community types align closely with soil biogeochemistry (indicator species; see Sims et al. 2013, Astudillo-García et al. 2019, Hermans et al. 2020), or whether community members are redundant to one another, their identity uncoupled from their biogeochemistry (Frossard et al. 2011, Louca et al. 2018).”

METHODS
l144: Unfortunately, FigS2 doesn’t show any data, at least in my file.

RR et al: publishing team: please check that the supplementary data file S1 and supplementary word document S2 are both included.

l151: please include the projection type that you used in this map.

RR et al: WGS-84; now added to the Figure 1 caption

l191: At this point, the reader is not familiar with the identifier M3. I suggest either to reference the position where these identifiers are explained, or to shortly state what kind of site it was. e.g. restored site M3

RR et al: Whether or not that sample amplifies has nothing to do with treatments, so it is better to use its unique identifier to not mislead people. The text now reads “1 sample (noted as M.3 in the supplementary data file) was removed during denoising due to low sequence count”

I 201: Being new to this method, I would appreciate some more information on how this extraction works in the main text.

RR et al: These analyses were contracted through a third party lab.

l205: Please include a reference for your spectrometry.

RR et al: These analyses were contracted through a third party lab.

l241: Paradis and Schleip 2018 is not included in the reference list

RR et al: thank you

l263-264: this statement is not easy to understand. Above you stated that your environmental data didn’t show a relationship between geography and soil variables but here you mention short environmental gradients. Please explain more carefully.

RR et al: The environmental gradients for a regional scale study are “short” compared to a continental scale study. We revised the sentence to: “Unlike CCA, which assumes a unimodal distribution of species along gradients, CAP assumes a linear distribution of species along gradients, which is well suited to environmental gradients at the regional scale (i.e. southeastern Massachusetts).”

RESULTS
l316 + l 337(+figure caption of fig 3): Adding up both percentages in fig. 3A/B I am not getting the 45% /44% mentioned in the text (36.5/28%). Whether this is due to the forward selection of particular variables or due to other reasons, please explain more carefully.

RR et al: the variation explained by the CAP model is higher than the variation explained by adding up the two axes, which is expected. The two axes represent the 2 best fitting gradients, but the model incorporates all the explanatory power of up to 13 variables.

Table S1 is missing many values for sample ER.2.5: It is not clear, however, if this is a real artefact, or if it is only missing by mistake. If values were not obtained for this sample, plese state something like n.d. in the excel file.

RR et al. That is correct- the sample is missing.

l350-352: In order to complete this information, I suggest to include two columns indicating the importance of each variable for microeucariotes/procaryotes, and to shorten the table caption accordingly.

RR et al. Thanks for the suggestion. It is not our intention to rank variables in order of importance, but rather to highlight how the most important variables respond to treatments. However, we did shorten the caption as recommended.

Fig. 4: I really like those graphs, they are very informative! However, you should stay consistent in what you are labelling and what you don’t. Please include Methanobacteria labeling that is missing in the graph depicting differences between natural and restored areas. Other labels of significant differences are also missing (same in fig. 5) and I am wondering why. Were they not as important or did you choose to label only the once with a certain log2 ratio of median proportions?

RR et al. Labels are somewhat arbitrary, but that is ok because this figure is just a visualization tool. The captions for the heat trees now read: “Colored labels showcase example taxa that were consistently more (teal) or less (orange) abundance in natural sites, or displayed variation across the four heat trees”.Taking note of the reviewer’s comment, we also removed most of the labels from Fig. 4 and Fig 5, so that there are only a handful of labels on each heat tree.

l379: …whereas taxa that are more abundant in retired, flooded, and restored… here, farm sites are missing from the description.

RR et al. corrected!

DISCUSSION
l391-392: Wasn’t this class (kathablepharidae) MORE abundant in flooded and restored sites (orange) as compared to natural sites? If this is the case, they seem to represent a species that gains importance in succession stages such as flooded and restored sites, but is not as important in the natural reference sites. Please correct also in the discussion.

RR et al. Yes, now corrected

Fig. 5: Nucletmycea are labelled as Nucleomycea in some graphs, please correct.

RR et al. addressed, thank you!

l408: This procedere has not been described in the methods. How did you define “standouts”?

RR et al. Revised to “While no individual prokaryotic genera showed any trends during data exploration, the eukaryote dataset showed a strong pattern..”
